# Gastroprotective and Antiulcerogenic Impacts of Pomegranates ( *Punica granatum* ) Peel Extract: In Vivo and In Vitro Study

**DOI:** 10.1002/fsn3.71411

**Published:** 2025-12-30

**Authors:** Talat M. Hashem, Manal A. Almughamisi, Ameerah M. Almaski, Suha H. Abduljawad, Adel A. Hassan

**Affiliations:** ^1^ Makkah Regional Laboratory Ministry of Health Makkah Saudi Arabia; ^2^ Department of Clinical Nutrition, Faculty of Applied Medical Sciences Taibah University Medina Saudi Arabia

## Abstract

Some natural items' anti‐ulcer properties were studied as possible alternative medications, most likely because of their efficacy and safety. Omeprazole is a cornerstone in the treatment of gastric ulcers, not only promoting ulcer healing but also preventing recurrence by reducing gastric acid secretion and aiding in mucosal regeneration. Pomegranate has been suggested as a potential protective food supplement to attenuate several pathological disorders such as hepatotoxicity, nephrotoxicity, cardiotoxicity, and tumors because of its rich composition of bioactive substances that provide antioxidant and anti‐inflammatory roles. The present work aimed to evaluate the gastroprotective and antiulcerogenic properties of Pomegranates peel extract by comparing it with Omeprazole. The phenolic content of Pomegranates peel extract was determined by using HPLC. Further in vitro investigations of Pomegranates peel extract, as anti‐inflammatory and anti‐ *Helicobacter pylori* activities, were carried out. The in vivo animal study included four groups of albino rats that were used in this study (control g., indomethacin ulcerated g., indomethacin + Omeprazole group, and indomethacin + Pomegranates peel extract group). In vivo studies included measuring gastric juice volume, biochemical evaluation of gastric free and total acidity, macroscopic ulcer index scoring, and microscopic histopathological examinations. Results exhibited that Pomegranates peel extract was rich in phenolic compounds and recorded significant anti‐inflammatory and anti‐ *H. pylori* activities. The in vivo animal study showed that both omeprazole and pomegranate peel extract achieved ameliorative gastroprotective and antiulcerogenic impacts against pathological alterations induced by indomethacin as the gastric injury decreased, and acidity improved, the gastric tissue returned to normal, and the ulcers healed. In conclusion, pomegranate peel extract accelerates gastric ulcer healing and shows anti‐ *H. pylori* and anti‐inflammatory activities.

## Graphical abstract

This study evaluated the gastroprotective potential of Pomegranate peel extract compared to Omeprazole. The extract demonstrated significant in vitro anti‐ *H. pylori* and anti‐inflammatory activities. In vivo experiments on indomethacin‐induced rats revealed that the extract effectively reduced gastric acidity and ulcer indices while restoring gastric mucosal histology, suggesting it is a promising natural therapeutic agent for gastric ulcers.

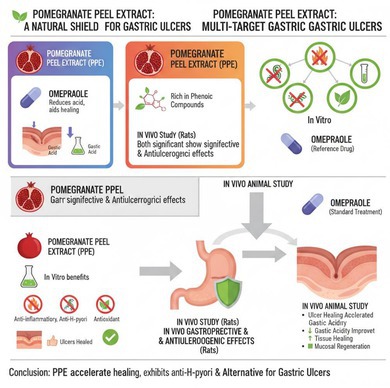

## 1 Introduction

Stomach ulcers are prevalent gastrointestinal conditions marked by a disruption in the mucosal barrier, leading to the degeneration and erosion of the gastric mucosa, which can extend to the muscularis mucosa and may penetrate the submucosa. Several factors contribute to ulcer formation (Alazzouni et al. 2020; Nabil et al. 2021). The development of stomach ulcers has been associated with various mechanisms, including impaired cell proliferation, oxidative stress, infiltration by neutrophils, overexpression of inflammatory mediators, and the release of pro‐inflammatory cytokines (Al Asmari et al. 2015). Additionally, the pathogenicity of gastric ulcers is primarily linked to an imbalance between the protective mechanisms (such as cellular regeneration, cell turnover, growth factors, and the production of mucin, bicarbonate, prostaglandins, and nitric oxide) and the harmful factors (such as *H. pylori* infection and heightened secretion of pepsin and gastric acids). Ulcer formation may also be associated with disturbances in microcirculation, leading to ischemia and reduced blood and oxygen supply to the gastric mucosa (Al Asmari et al. 2015; El‐Azab et al. 2018). The healing process of gastric ulcers is a complex regenerative process involving cell migration, proliferation, re‐epithelialization, and the restoration of underlying connective tissue, including the blood vessels, muscle layers, glandular structures, angiogenesis, vasculogenesis, and matrix deposition, culminating in scar formation (Ajayi et al. 2016; Sheir and Ahmed 2022).

Omeprazole is a highly effective treatment for gastric ulcers, a condition characterized by the erosion of the stomach lining due to excessive gastric acid secretion. As a proton pump inhibitor, omeprazole reduces gastric acid production by inhibiting the H^+^/K^+^‐ATPase enzyme in the parietal cells of the stomach. This reduction in acid secretion creates an environment that facilitates the healing of gastric ulcers, promotes mucosal regeneration, and prevents further ulcer formation (Rudra et al. 2022). While omeprazole is widely recognized for its effectiveness in treating gastric disorders, it is important to be aware of its potential side effects and long‐term risks. Most side effects are mild and transient, but in some cases, prolonged use or high doses can lead to more serious complications (Haastrup et al. 2018). However, most of current treatment options for gastric ulcers are often accompanied by side effects and a high rate of recurrence.

The potential anti‐ulcer properties of various botanicals, including plants, herbs, fruits, dietary antioxidants, and vegetables, are being explored as alternative therapies due to their safety, efficacy, and the presence of bioactive phytochemicals (such as flavonoids, terpenoids, and saponins), along with their availability and low cost (Ajayi et al. 2016; El‐Azab et al. 2018; Sheir and Ahmed 2022).

Pomegranates ( *Punica granatum* ) are increasingly used for their flavor and nutritional value. Pomegranates have been extensively studied in recent years for their numerous health benefits as it exhibits potent anticancer, antioxidant, and anti‐inflammatory, cardioprotective, and cognitive properties, primarily attributed to their rich content of bioactive compounds, as, dietary fiber, vitamins, minerals, polyphenols, flavonoids, and tannins, which all work synergistically to promote health and prevent disease (Bookheimer et al. 2013; Turrini et al. 2015; Deng et al. 2017; Sahebkar et al. 2017; Vučić et al. 2019; Singh et al. 2018; Liu et al. 2019; Mo et al. 2022; Alami et al. 2023).

Regular consumption of pomegranate has been linked to cardiovascular benefits as it significantly reduces systolic and diastolic blood pressure, suggesting its potential role in hypertension management (Bookheimer et al. 2013). Furthermore, pomegranate juice supplementation enhanced memory performance and increased plasma antioxidant levels in middle‐aged and older adults (Sahebkar et al. 2017). Pomegranate extract can modulate inflammatory pathways, thereby reducing chronic inflammation associated with diseases such as arthritis (Vučić et al. 2019).

Pomegranates peel making up 26%–30% of the fruit's weight. However, the by‐products from fruit processing, like peels and seeds, can cause environmental pollution if not managed properly. However, many reports showed that these by‐products offer various health benefits (Liu et al. 2019; Mo et al. 2022). Treatment of *Helicobacter pylori* ( *H. pylori* ) is essential for reducing inflammatory responses of the stomach mucosa and stopping the development of stomach disorders (Elbestawy et al. 2023). Despite the fact that antibiotic‐based treatments have been quite successful in eliminating *H. pylori* , problems have surfaced, including antibiotic resistance, medication toxicity, adverse implications, failure to adhere, inappropriateness, and disturbance of the gut microbiota. To control *H. pylori* , new safe treatments are desperately needed (Deng et al. 2024).

In this context, this study aimed to investigate the gastroprotective, antiulcerogenic, and ulcer healing activities of Pomegranates ( *Punica granatum* ) compared with Omeprazole in an attempt to clarify a novel gastroprotective botanical agent.

## 2 Materials and Methods

### 2.1 Omeprazole

Capsules of omeprazole were purchased [Pepzol MR is the trade name; Hikma Pharma S.A.E company]. Each capsule of omeprazole (20 mg) was dissolved in saline solution (Sigma Aldrich Company, St. Louis, USA). A dose of (20 mg/kg BW, once a day for 15 days) was applied (Ketuly et al. 2011).

### 2.2 Ulcer Induction

Indomethacin was bought from (Sigma, St. Louis, MO, USA). The approach outlined by Sayanti et al. (2007) was used to generate gastric ulcers in the animals. In short, rats were given 30 mg/kg body weight of indomethacin orally as a single dosage. 24 h before ulcer development, rats were given free access to water but were deprived of food. Four hours after indomethacin was administered, different levels of ulceration appeared.

### 2.3 Pomegranate Rind Powder Extraction

The peels of pomegranates were allowed to dry at ambient temperature. Dried herbs were then ground into a fine powder. Next, 50 g of peels were ground into a powder, macerated in 0.5 L of 70% ethanol in a secured container, and left to sit for 48 h at room temperature. For traditional extraction, the powder and extract were put in a sonicator set to 40°C for 60 min. Rota vapor was then used to filter and condense this extract under pressure at 40°C, yielding 19.0 g of crude extract (Tang et al. 2010).

### 2.4 Separation of Bioactive Molecules of Pomegranates Peel Extract Using HPLC

“The HPLC test was conducted using an Agilent 1260 instrument, and the separation was performed using a Zorbax Eclipse Plus C8 column (4.6 × 250 mm i.d., 5 μm). Water (A) and 0.05% trifluoroacetic acid in acetonitrile (B) at a flow rate of 0.9 mL/min made up the mobile phase. The following is the sequential programming of the mobile phase in a linear gradient: The multi‐wavelength detector recorded at 280 nm for the following times: 0 min (82% A); 0–1 min (82% A); 1–11 min (75% A); 11–18 min (60% A); 18–22 min (82% A); and 22–24 min (82% A). For each specimen solution, the injection volume was 5 μL, and the column temperature was maintained at 40°C (Burton‐Freeman et al. 2017).”

Similar to previous publications (Lu and Yuan 2008; Man et al. 2022), the current study identified around 15 phenolic compounds in pomegranate peel that were measured using standards, including punicalagin, ellagic acid, gallocatechin, punicalin, catechin, and corilagin. El‐Hadary and Ramadan (2019) used HPLC to identify 23 phenolic components in the peel of Egyptian pomegranates. Gallic acid, coffeic acid, ellagic acid, rosmarinic acid, ferulic acid, coumaric acid, vanillin, and chlorogenic acid were the eight phenolic chemicals that were identical in this investigation. However, the phenolic acid profiles of pomegranates and their levels may differ depending on the geographic conditions where they are cultivated (Elfalleh et al. 2011).

### 2.5 Assessment of Anti‐ *H. pylori* and Detection of Minimum Inhibition Concentration (MIC) and Minimum Bactericidal Concentration (MBC) for the Pomegranates Peel Extract

To assess the extracted substance's impact on *H. pylori*, we were gratefully given a strain of *H. pylori* (ATCC 43504) by the National research center. Using the well agar diffusion method, the anti‐ *H. pylori* qualities were examined in vitro. A 100 μL solution of *H. pylori* (1.0 × 10^8^ CFUs/mL) was added to Mueller Hinton agar dishes that contained 10.0% sheep blood. A 6 mm circular cut was then aseptically bored using a sterile cork borer. After that, 100 μL of the sample at the specified level was added to the well. The antibiotics amoxicillin (0.06 mg/mL) and clarithromycin (0.06 mg/mL) served as positive controls, and DMSO served as the negative control. Following a 72‐h incubation period at 35°C in a moist microaerophilic apparatus, the dimensions of the inhibitory area were assessed (Grande et al. 2021). Detection of Minimum inhibition concentration (MIC) and minimum bactericidal concentration (MBC) for the Pomegranates Peel extract were carried out according to Huang et al. (2021).

### 2.6 Denaturation Method for Characterization of Anti‐Inflammatory Impact

To evaluate the anti‐inflammatory activity of Pomegranates Peel extract, 50 μL of the sample was combined with 450 μL of a 1% aqueous solution of bovine serum albumin at several concentrations ranging from 1.56 to 200 μg/mL. The solution's pH was lowered to 6.3 by adding a little amount of 1 N hydrochloric acid. These samples were incubated at room temperature for 20 min before being heated to 55°C for 30 min in a water bath. After the samples were cooled following the heating process, the absorbance at 660 nm was measured using a Biosystem 310 plus spectrophotometer. Diclofenac sodium was used as the reference drug for comparison. Dimethyl sulfoxide (DMSO) served as the experiment's negative control (M et al. 2023).

### 2.7 Animals and Experimental Design

In this investigation, twenty‐four male albino rats in good health, weighing between 170 and 190 g, were chosen. For a week before the studies, the animals were kept in standard conditions in the laboratory (25°C–27°C, 60% humidity, and a 12‐h light/dark cycle) to optimize performance and exclude out disease. They were also given free access to a standard meal and water. The King Abdulaziz University animal ethics committee approved the study, which was carried out in compliance with international regulations and guidelines (NIH guidelines for the Care and use of laboratory animals, NIH publication No. 85‐23, 1985; EEC Council directives 86/609, OJL 358, 1 December, 12, 1987).

### 2.8 Experimental Groups

Rats were divided into 4 groups, 6 rats in each.

*Group I*: Negative control group: Rats of this group received normal saline orally for 2 weeks.

*Group II*: Positive control group: Rats treated with indomethacin as a single oral dose (30 mg/kg body weight) to induce ulcer.

*Group III*: Omeprazole group (OG): Rats received indomethacin followed by Omeprazole (20 mg/kg BW, once daily for 15 days) for 2 weeks.

*Group IV*: Pomegranate peel extract group (PG): Rats received indomethacin as a single oral dose of indomethacin, followed by a pomegranate peel extract suspension at a dose of 75 mg/kg orally for 2 weeks. This dose is consistent with the dose that people usually use (one tablespoon), which contains a concentration of 300–350 mg/kg of anti‐inflammatory components (Jumaa Jandal and Naji 2021).

All rats were killed by cervical dislocation at the end of the experiment, and their stomachs were carefully dissected out.

### 2.9 Assessment of Anti‐Ulcer Activity

#### 2.9.1 Gastric Volume Estimation

Four hours after the induction of gastric ulcers, the stomachs were carefully dissected out. The contents of the stomach were collected into a graduated measuring cylinder to determine the volume of gastric fluid.

#### 2.9.2 Determination of Gastric Free and Total Acidity

Using Topfer's reagent and phenolphthalein as an indicator, the gastric juice was titrated with 0.01 N NaOH to evaluate its free and total acid content. The results were expressed as Meq/L/24 h (Jainu et al. 2006; Wang et al. 2007). In the lab, a burette was set up, and 0.01 N NaOH was made and added to it. The aliquot of stomach content in the simple bottles was supplemented with 50 mL of distilled water. To compensate for the free acid, three drops of Topfer's reagent were added to 25 mL of gastric juice that had been pipetted into a beaker. After titrating the NaOH within the burette against the beaker's acidic solution, the mixture was watched until it became yellow. It was observed how much alkali was utilized, which is equivalent to the free acidity.

### 2.10 Estimation of Gastric Ulcerative Index Changes

The Takagi et al. (1969) technique was used to measure the ulcerative index. The stomach was opened along the larger curvature for a brief moment. Water from the flowing tap was used to cleanse the stomach. The ulcerative area was then counted by placing it on a flat wooden plate. The following formula was used to calculate the ulcer index:

Ulcer Index=10/X

where X = Total mucosal area/Total ulcerated area.

Ulcer protection inhibition (%) was calculated using the formula:

$$\text{Ulcer protection}\;\left(\%\right)=100-\mathrm{Ut}/\mathrm{Uc}\times 100$$

where Ut = Ulcer index of treated group; Uc = Ulcer index of control group.

### 2.11 Histological Studies

After being fixed in Bouin solution for about 24 h, small stomach specimens from various experimental groups were washed, dried, cleaned in xylene, and saturated with parablast paraffin wax, then blocking. For microscopic analysis of stomach injuries and any regeneration process, five μm thick slices were made using a rotary microtome and then stained with Hematoxylin and Eosin (H&E) stain (Bancroft and Gamble 2008).

### 2.12 Statistical Analysis

The mean ± standard error of means (SEM) was used to express all the biochemical data. Sigma Stat Version‐3.5 software was used to analyze the data using Turkey's multiple range tests. Statistical significance was defined as a probability value of *p* < 0.05. Three iterations of the microbiological experiments were conducted, and the results were all presented as means ± standard deviation (SD). The findings were drawn using Graph Pad Prism V6.

## 3 Results and Discussion

### 3.1 Assessment of Anti‐ *H. pylori* MIC and MBC

**FIGURE 1:**
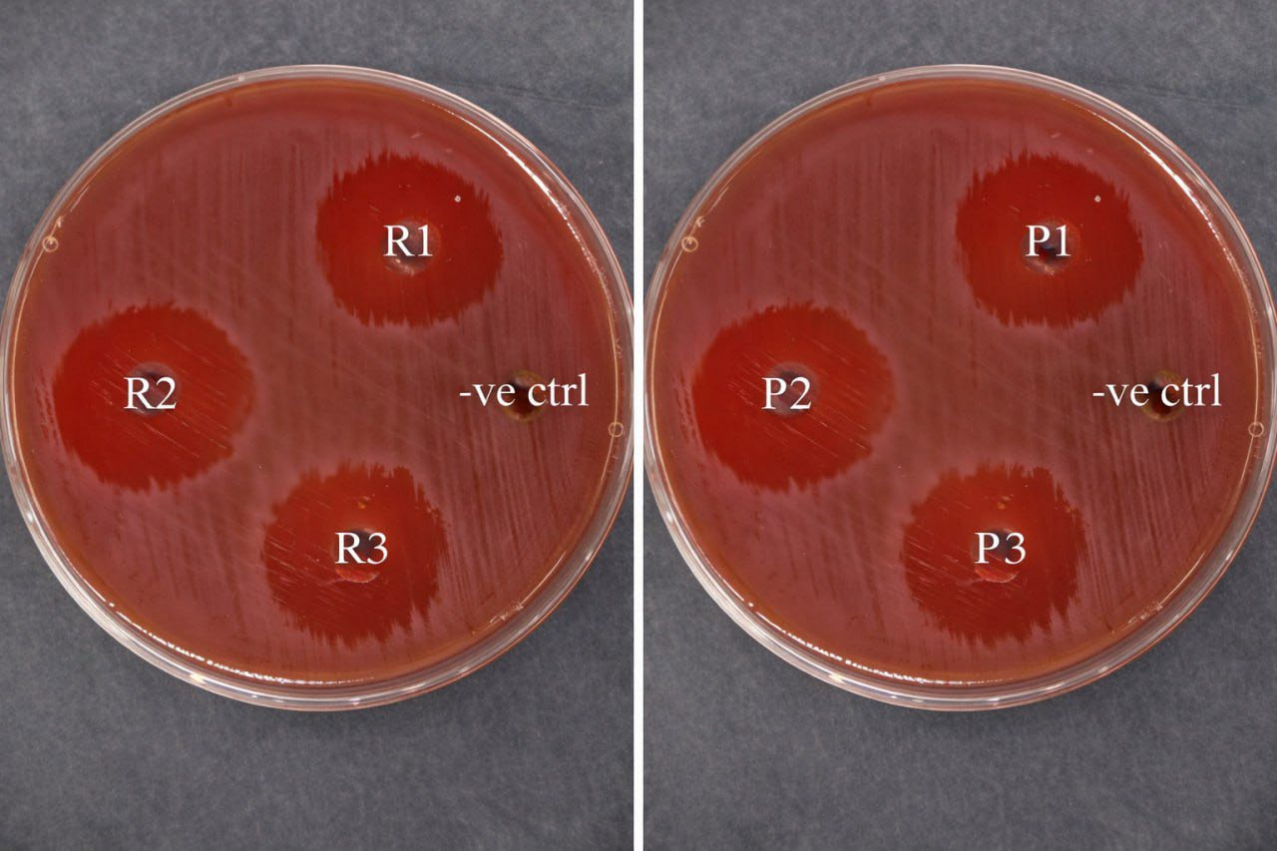
Determination of anti‐ *H. pylori* of the produced pomegranate peels extract (left plate: Contained three replicates of standard drug (R1‐R3); right plate: Contained three replicates of pomegranate peels extract (P1‐P3); −ve ctrl in both plates refer to negative control).

In this work, as shown in Figure 1 and Table 1; detection of minimal inhibitory level and minimal bactericidal level for the pomegranate peels extract showed an inhibition zone of 23.0 ± 0.1 which was at the same level for the standard drug. Besides, the level of 31.25 ± 0.1 μg/mL was determined as MIC and MBC for pomegranate peels extract. Besides, MBC/MIC index was reported at level = 1, revealing its bactericidal action against *H. pylori* . The present results revealed the promising bactericidal impact of pomegranate peel extract towards *H. pylori* . Recent studies have explored the potential of pomegranate in combating *H. pylori* , a bacterium linked to various gastric disorders, including ulcers and gastritis. Research indicates that pomegranate peel ethanol extract (PPEE) exhibits significant activity against *H. pylori* , with a minimum inhibitory concentration (MIC). Additionally, PPEE demonstrates notable urease inhibitory activity, an enzyme crucial for *H. pylori* survival in the acidic gastric environment. In vivo studies on rats have shown that oral administration of PPEE significantly reduces *H. pylori* ‐induced gastritis and decreases associated inflammation (Mayyas et al. 2021). The anti‐ *H. pylori* effects of ethnomedicine and its natural products have been the subject of several in vitro and in vivo investigations. In terms of incidence, Sisto et al. (2016) showed that artemisone and artemisinin derivatives were effective against *H. pylori* in vitro. Furthermore, myricetin is a naturally occurring chemical that inhibits the morphological change of *Helicobacter pylori* from spiral to coccoid forms, increasing antibiotic resistance, according to Krzyżek et al. (2021). Bacterial mortality can result from gallic acid and ellagic phenolic substances precipitating membrane proteins and preventing enzyme function (Monika et al. 2022).

**TABLE 1:** Detection of inhibition diameter (mm) of pomegranate peel extract, MIC, MBC and MBC/MIC index (Data are reported as means ±0.1).

Sample code	Inhibition zone (mm)	MIC (μg/ml)	MBC (μg/ml)	MBC/MIC index#
Control	24.0 ± 0.1	15.62 ± 0.1	31.26 ± 0.1	2
Pomegranate peels Extract	23.0 ± 0.1	31.25 ± 0.1	31.25 ± 0.1	1

*Note:* The MBC/MIC of the specimen ≤ 4 illustrate their bactericidal impact towards *H. pylori* , while the MBC/MIC of the specimen > 4 illustrates their bacteriostatic activity.

### 3.2 Denaturation of Anti‐Inflammatory Activity

**FIGURE 2:**
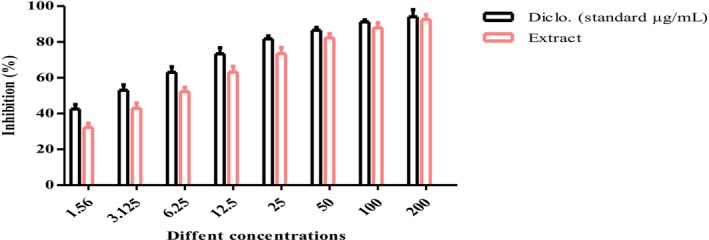
Illustration of anti‐inflammatory role for pomegranate peels extract using denaturation test (results were drawn as means ± SD).

In this work, pomegranate peels extract exhibited marked anti‐inflammatory impact. Denaturation test was applied for determination of the anti‐inflammatory role of pomegranate peels extract at IC50 = 4.16 ± 0.6 μg/mL. While the anti‐inflammatory level for the diclofenac standard was reported at IC50 = 1.58 ± 0.1 μg/mL as drawn in (Figure 2). Current reports proved the possible role of pomegranate in decreasing oxidative stress, inflammation, apoptosis, and oral mucosal bacterial proliferation (Sayed et al. 2022; Dathan et al. 2025; Silla et al. 2025). By altering COX‐2 regulation in the viable epidermis, pomegranates may offer a unique way to reduce the discomfort and inflammation linked to a variety of skin disorders, such as cold sores and herpetic stromal keratitis (Houston et al. 2017). Furthermore, Punicalagin from pomegranate was found to ameliorate TNF‐α/IFN‐γ‐induced inflammatory responses (Huang et al. 2024).

### 3.3 Biochemical Results

As shown in Table 2, Indomethacin induced significant accumulation in gastric fluid volume, with significant increase of free acidity and total acidity when compared with the control group. Omeprazole treated group exhibited significant reduction in gastric volume increase in gastric fluid volume, free acidity and total acidity when compared with indomethacin group. Treating rats with Pomegranate peel excretion induced significant improvement of all ulcerative parameters as gastric fluid volume, free acidity, and total acidity when compared with the indomethacin group, while there was an insignificant difference between the Omeprazole and Pomegranate peel excretion treated groups.

**TABLE 2:** Alteration in gastric fluid volume, free acidity, total acidity, ulcerative index and % inhibition among experimental groups (*n = 6*; Mean ± SE; *p* < 0.05).

Group (mg k g^−1^)	Gastric fluid volume (mL 100 g^−1^)	Free acidity (mEq L^−1^)	Total acidity (E = mEq L^−1^)	Ulcerative index	% inhibition
I. Control	1.46	32.22	59.29	0.0	0.0
II. Indomethacin	2.86^a^	62.39^a^	109.15^a^	5.1^a^	0.0
III. Omeprazole	1.33^b^	27.41^b^	58.51^b^	1.1^b^	78.40
V. Pomegranate	1.28^b^, ^c^	26.94^b^, ^c^	60.16^b^, ^c^	1.3^b^, ^c^	74.51^c^

^a^ Significant increase when compared with control group.

^b^ Significant decrease when compared with indomethacin group.

^c^ Insignificant difference when compared with Omeprazole group (Turkey test).

### 3.4 Histological Results

**FIGURE 3:**
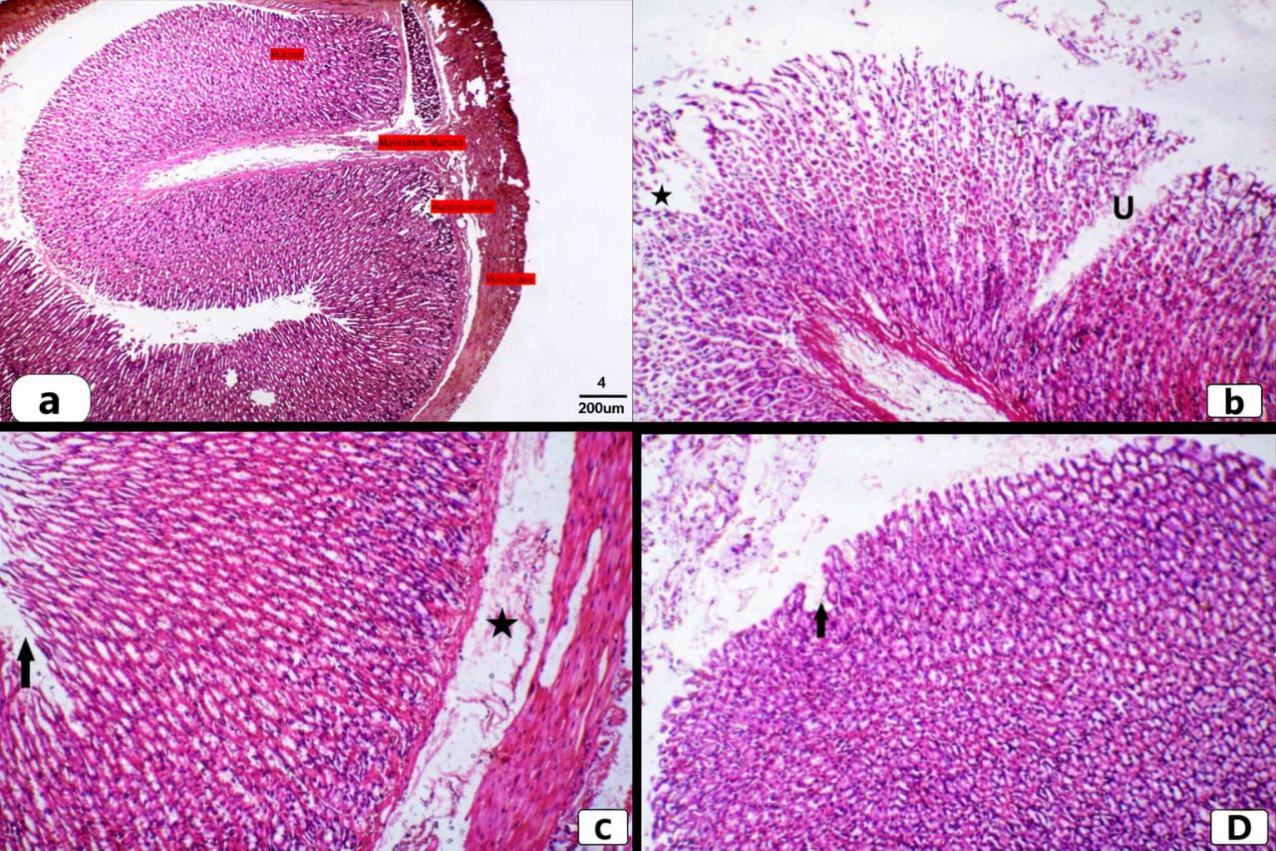
Microscopic examination of H&E stained sections showing histopathological alterations in gastric tissue among different groups. (a) Section from a control rat showing normal histological structure of stomach and its layers mucosa, submucosa, muscularis mucosa, and serosa (X4). (b) Section from a rat from indomethacin ulcerated group, showing deep gastric ulcer (u) eroding through the mucosa, and degeneration (star) (X10). (c) Section from a rat from omeprazole group showing improvement of gastric tissue with healed gastric ulcer (arrow), and slight submucosal oedema (star) (scale bar 100 μm) (X10). (d) Section from rat treated with pomegranate peel extract showing like normal gastric tissue with normal gastric layers and healed gastric ulcer (arrow) (X10).

Hematoxylin and Eosin‐stained slides from the control group showed the normal histological structure of the stomach layers (mucosa, submucosa, and musculosa) when examined under a microscope. The tall mucus‐secreting columnar epithelium lines the stomach mucosa, which is bordered with the long tubular gastric (oxyntic) glands and extends as far down as the muscularis mucosa. The lamina propria divided the small, numerous, straight, and perpendicular gastric glands from one another. The glands opened onto the surface through tiny gastric pits. The second layer is the submucosa, which is made up of blood vessels and smooth muscle fibers in loose connective tissue (CT). Muscularis, the third layer, is made up of circular and longitudinal muscle bundles. The serosa, which is the last layer, is made up of blood vessels and C.T. (Figure 3a).

The administration of indomethacin caused gastric ulcer and damage by comparing with the control group, as evidenced by severe cellular degenerative changes, necrosis and submucosal oedema, as well as neutrophil and lymphocyte infiltration in the lamina propria and submucosa, which caused an erosion of the superficial epithelial cells in some areas of the gastric mucosae and appeared to be acute ulcers, which were confirmed by morphometric assessments that showed a significant increase in the macroscopic evaluation of the ulcer index when compared to the control group (Figure 3b; Table 1). Many studies reported Indomethacin as gastric ulcer inducing agents that increase acidity, and induces several histopathological alterations in gastric tissue (Sayanti et al. 2007; Nabil et al. 2021). When compared to the Indomethacin‐ulcerated group, the omeprazole‐treated group demonstrated a notable improvement in gastric tissue, as evidenced by a significant decrease in the ulcer index and gastric tissue injury. Additionally, all of the animals in this group didn't display any degenerative changes, necrotic areas, or inflammatory infiltration (Figure 3c; Table 1).

The present results are in accordance with Mohan Prasad et al. (2024) who reported that omeprazole significantly improves the healing of peptic ulcers by reducing gastric acidity, which is a key factor in ulcer formation and persistence. The researchers found that patients receiving omeprazole showed faster recovery and reduced recurrence of ulcers compared to those receiving alternative treatments. Moreover, Rudra et al. (2022) indicated that omeprazole can reduce the levels of pro‐inflammatory cytokines in the gastric mucosa via preventing stress‐induced gastric ulcer by direct inhibition of MMP‐2/TIMP‐3 interactions thus contributing to the protection of the gastric lining and aiding in the healing process of ulcers. A significant study by Demertzis et al. (2006) demonstrated that omeprazole not only accelerates the healing process of gastric ulcers but also prevents ulcer recurrence by maintaining a neutral pH in the stomach, which is essential for optimal healing.

Rats treated with pomegranate peel extract demonstrated a significant improvement in gastric histoarchitecture with the classical histological structure of normal gastric layers (mucosa, submucosa, and musculosa). Additionally, desquamated surface epithelial cells were healed, with no histopathological changes other than slight submucosal oedema in some examined sections. This was confirmed by morphometric assessments that revealed a significant reduction in macroscopic evaluation of ulcer index when compared to the indomethacin group. Additionally, there was an insignificant difference with the Omeprazole treated group (Figure 3d; Table 1). Regarding ulcerative index and ulcerative inhibition index (%), both of Omeprazole and Pomegranate peel excretion induced marked activities, without any significant difference.

Instead of using potentially harmful chemical treatments to treat stomach ulcers, recent research has tended to employ natural alternatives. As a naturally occurring anti‐ulcer agent with high phenolic component contents, Sheir and Ahmed (2022) examined the function of several bee honey varieties (Clover, Sidr, and Samar honey) in the healing of stomach ulcers in albino rats. By lowering MPO and PGE2 levels, gastric juice volume, pH, and acidity, as well as by enhancing mucus contents, wound contraction and epithelialization through the regulation of iNOS, HSP47, and VEGF immunohistochemical expression, all varieties of honey under investigation demonstrated a protective effect on gastric mucosal damage. Additionally, honey demonstrated *anti‐H. pylori* action, decreased hemosiderin deposits, and accelerated cell migration in wound scratch assays (Sheir and Ahmed 2022). The ulcer inhibition (%) of *Punica granatum* at 500 and 250 mg/kg was 84.60% ± 5.48% and 42.87% ± 7.14%, respectively, according to Muhialdin et al. (2023). These values were significantly higher than those of the omeprazole group (24.50% ± 6.35%; *p* = 0.0001). Pomegranate also reduced the stomach index and improved gastric tissue integrity (Muhialdin et al. 2023). In contrast, omeprazole exhibited a much higher inhibition percentage in the present study (78.40%). This difference likely reflects variations in ulcer induction models, since the model used in our study is more responsive to proton‐pump inhibition, whereas that used by Muhialdin et al. involved stronger oxidative and inflammatory injury. Differences in dosage, formulation, administration route, treatment duration, and animal strain sensitivity may further explain the discrepancy. These methodological distinctions indicate that the higher omeprazole efficacy in our findings is consistent with the experimental conditions employed.

The possible therapeutic benefits of pomegranates ( *Punica granatum* ) for stomach ulcers have been the subject of several investigations. Pomegranate extracts have been shown to improve the levels of anti‐oxidative enzymes, lower tissue lipid peroxidation, and considerably inhibit gastrointestinal ulcers. The fruit's strong antioxidant content, which aids in scavenging free radicals and protecting the stomach mucosa, is responsible for these benefits (Cherrada et al. 2024). Mohamed and Mabrok (2022), found that administration of pomegranate peel extract to rats with induced gastric ulcers resulted in a significant decrease in ulcer index and gastric acidity. The pomegranate extract also promoted healing by increasing mucus production and enhancing antioxidant status. Pomegranate's antioxidant properties are attributed to its ability to reduce lipid peroxidation, boost the activities of endogenous antioxidants including catalase (CAT) and superoxide dismutase (SOD), and neutralize reactive oxygen species (ROS). Additionally, the inhibition of pro‐inflammatory cytokines like TNF‐α and IL‐1β, together with the encouragement of stomach mucosal protection agents, mediate the anti‐inflammatory actions of pomegranates. According to histological investigations, pomegranate extract reduces the severity of ulcers and maintains the architecture of stomach tissue (Zamanian et al. 2025). TNF‐α expression is up‐regulated in conjunction with stomach ulceration. According to earlier research, pomegranate polyphenolic components reduce the expression of pro‐inflammatory cytokines like TNF‐α. These results show that via lowering the expression of the COX‐2 and TNF‐α genes, pomegranate peel powder has antiulcer and anti‐inflammatory properties (Ahmed et al. 2020; Serafim et al. 2020).

## 4 Conclusion

Because of its high level of phenolic compounds, the present study concluded that pomegranate peel extract had anti‐inflammatory, gastroprotective, and anti‐ *H. pylori* activities in addition to accelerating the healing of stomach ulcers. These results demonstrate the potential of pomegranate as a natural alternative treatment for stomach ulcers, which needs more research into its modes of action and the best possible therapeutic combinations.

## Funding

No funding sources.

## Conflicts of Interest

The authors declare no conflicts of interest.

## Data Availability

All data generated or analyzed during this study are included in this published article.

## References

[fsn371411-bib-0001] Ahmed, I. , M. A. Elkablawy , D. S. El‐Agamy , A. A. Bazarbay , and N. Ahmed . 2020. “Carvedilol Safeguards Against Aspirin‐Induced Gastric Damage in Rats.” Human & Experimental Toxicology 39: 1257–1267. 10.1177/0960327120918306. 32295429

[fsn371411-bib-0002] Ajayi, A. F. , A. M. Folawiyo , and T. A. Salami . 2016. “Ulcer Healing Mechanism of Ethanolic Extract of *Talinum triangulare* in Male Wistar Rats.” Pharmaceutical and Biosciences Journal 4: 41–50. 10.20510/UKJPB/4/I4/110647.

[fsn371411-bib-0003] Al Asmari, A. , H. Al Shahrani , N. Al Masri , A. Al Faraidi , I. Elfaki , and M. Arshaduddin . 2015. “Vanillin Abrogates Ethanol Induced Gastric Injury in Rats via Modulation of Gastric Secretion, Oxidative Stress and Inflammation.” Toxicology Reports 3: 105–113. 10.1016/j.toxrep.2015.11.001. 28959528 PMC5615375

[fsn371411-bib-0004] Alami, M. , K. Boumezough , A. Khalil , et al. 2023. “The Modulatory Bioeffects of Pomegranate (*Punica granatum* L.) Polyphenols on Metabolic Disorders: Understanding Their Preventive Role Against Metabolic Syndrome.” Nutrients 15, no. 23: 4879. 10.3390/nu15234879. 38068738 PMC10707905

[fsn371411-bib-0005] Alazzouni, A. S. , A. S. Fathalla , M. S. Gabri , M. A. Dkhil , and B. N. Hassan . 2020. “Role of Bone Marrow Derived‐Mesenchymal Stem Cells Against Gastric Ulceration: Histological, Immunohistochemical and Ultrastructural Study.” Saudi Journal of Biological Sciences 27, no. 12: 3456–3464. 10.1016/j.sjbs.2020.09.044. 33304156 PMC7715057

[fsn371411-bib-0029] Ameena, M ., M.Arumugham , K. Ramalingam , S. Rajeshkumar , and R. Shanmugam . 2023. “Evaluation of the Anti‐Inflammatory, Antimicrobial, Antioxidant, and Cytotoxic Effects of Chitosan Thiocolchicoside‐Lauric Acid Nanogel.” Cureus 15, no. 9: e46003. 10.7759/cureus.46003. 37900405 PMC10600588

[fsn371411-bib-0006] Bancroft, J. D. , and M. Gamble . 2008. Theory and Practice of Histological Techniques. 5th ed, 153. Churchill Livingstone.

[fsn371411-bib-0007] Bookheimer, S. Y. , B. A. Renner , A. Ekstrom , et al. 2013. “Pomegranate Juice Augments Memory and FMRI Activity in Middle‐Aged and Older Adults With Mild Memory Complaints.” Evidence‐Based Complementary and Alternative Medicine: Ecam 2013: 946298. 10.1155/2013/946298. 23970941 PMC3736548

[fsn371411-bib-0008] Burton‐Freeman, B. M. , A. K. Sandhu , and I. Edirisinghe . 2017. “Mangos and Their Bioactive Components: Adding Variety to the Fruit Plate for Health.” Food & Function 8: 3010–3032. 10.1039/c7fo00190h. 28612853

[fsn371411-bib-0009] Cherrada, N. , A. E. Chemsa , N. Gheraissa , et al. 2024. “Gastroprotective Efficacy of North African Medicinal Plants: A Review on Their Therapeutic Potential for Peptic Ulcers.” Food Science & Nutrition 12, no. 11: 8793–8824. 10.1002/fsn3.4536. 39619964 PMC11606823

[fsn371411-bib-0010] Dathan, P. , D. Nallaswamy , S. Rajeshkumar , S. Joseph , I. Shahin , and M. Tharani . 2025. “In Vitro Evaluation of Anti‐Inflammatory, Anti‐Oxidant Activity of Pomegranate Peel Extract Mediated Calcium Sulfate Nano Particles.” Medical Journal of Malaysia 80, no. Suppl 1: 44–51. 39773942

[fsn371411-bib-0011] Demertzis, K. , D. Polymeros , T. Emmanuel , K. Triantafyllou , P. Tassios , and S. D. Ladas . 2006. “Omeprazole Maintenance Therapy Prevents Recurrent Ulcer Bleeding After Surgery for Duodenal Ulcer.” World Journal of Gastroenterology 12, no. 5: 791–795. 10.3748/wjg.v12.i5.791. 16521197 PMC4066134

[fsn371411-bib-0012] Deng, R. , X. Chen , S. Zhao , Q. Zhang , and Y. Shi . 2024. “The Effects and Mechanisms of Natural Products on *Helicobacter pylori* Eradication.” Frontiers in Cellular and Infection Microbiology 14: 1360852. 10.3389/fcimb.2024.1360852. 38481665 PMC10933111

[fsn371411-bib-0013] Deng, Y. , Y. Li , F. Yang , et al. 2017. “The Extract From *Punica granatum* (Pomegranate) Peel Induces Apoptosis and Impairs Metastasis in Prostate Cancer Cells.” Biomedicine & Pharmacotherapy = Biomedecine & Pharmacotherapie 93: 976–984. 10.1016/j.biopha.2017.07.008. 28724216

[fsn371411-bib-0014] El‐Azab, N. E. , A. E. Mansy , A. M. El‐Mahalawy , and D. Sabry . 2018. “Comparative Study of the Therapeutic Effect of Pomegranate Alone or in Combination With Bone Marrow Mesenchymal Stem Cells on Experimentally Induced Gastric Ulcer in Adult Male Rats: A Histological and Immunohistochemical Study.” Egyptian Journal of Histology 41, no. 2: 150–166. 10.21608/EJH.2018.13838.

[fsn371411-bib-0015] Elbestawy, M. K. M. , G. M. El‐Sherbiny , and S. A. Moghannem . 2023. “Antibacterial, Antibiofilm and Anti‐Inflammatory Activities of Eugenol Clove Essential Oil Against Resistant *Helicobacter pylori* .” Molecules 28: 2448. 10.3390/molecules28062448. 36985419 PMC10058968

[fsn371411-bib-0016] Elfalleh, W. , N. Tlili , N. Nasri , et al. 2011. “Antioxidant Capacities of Phenolic Compounds and Tocopherols From Tunisian Pomegranate (*Punica granatum*) Fruits.” Journal of Food Science 76, no. 5: C707. 10.1111/j.1750-3841.2011.02179.x. 22417416

[fsn371411-bib-0017] El‐Hadary, A. E. , and M. F. Ramadan . 2019. “Phenolic Profiles, Antihyperglycemic, Antihyperlipidemic, and Antioxidant Properties of Pomegranate (*Punica granatum*) Peel Extract.” Journal of Food Biochemistry 43, no. 4: e12803. 10.1111/jfbc.12803. 31353600

[fsn371411-bib-0018] Grande, R. , S. Carradori , V. Puca , et al. 2021. “Selective Inhibition of *Helicobacter pylori* Carbonic Anhydrases by Carvacrol and Thymol Could Impair Biofilm Production and the Release of Outer Membrane Vesicles.” International Journal of Molecular Sciences 22: 11583. 10.3390/ijms222111583. 34769015 PMC8584244

[fsn371411-bib-0019] Haastrup, P. F. , W. Thompson , J. Søndergaard , and D. E. Jarbøl . 2018. “Side Effects of Long‐Term Proton Pump Inhibitor Use: A Review.” Basic & Clinical Pharmacology & Toxicology 123, no. 2: 114–121. 10.1111/bcpt.13023. 29658189

[fsn371411-bib-0020] Houston, D. M. , J. Bugert , S. P. Denyer , and C. M. Heard . 2017. “Anti‐Inflammatory Activity of *Punica granatum* L. (Pomegranate) Rind Extracts Applied Topically to Ex Vivo Skin.” European Journal of Pharmaceutics and Biopharmaceutics 112: 30–37. 10.1016/j.ejpb.2016.11.014. 27867111

[fsn371411-bib-0021] Huang, W. C. , C. J. Liou , S. C. Shen , et al. 2024. “Punicalagin From Pomegranate Ameliorates TNF‐α/IFN‐γ‐Induced Inflammatory Responses in HaCaT Cells via Regulation of SIRT1/STAT3 Axis and Nrf2/HO‐1 Signaling Pathway.” International Immunopharmacology 130: 111665. 10.1016/j.intimp.2024.111665. 38367463

[fsn371411-bib-0022] Huang, X. , Y. Liu , Z. Lin , et al. 2021. “Minimum Inhibitory Concentrations of Commonly Used Antibiotics Against *Helicobacter pylori* : A Multicenter Study in South China.” PLoS One 16, no. 9: e0256225. 10.1371/journal.pone.0256225. 34473713 PMC8412354

[fsn371411-bib-0023] Jainu, M. , K. Vijai Mohan , and C. S. Shyamala Devi . 2006. “Gastroprotective Effect of *Cissus quadrangularis* Extract in Rats With Experimentally Induced Ulcer.” Indian Journal of Medical Research 123, no. 6: 799–806. 16885602

[fsn371411-bib-0024] Jumaa Jandal, M. M. , and E. Z. Naji . 2021. “Study of the Effect of Ethanolic Extract of Pomegranate Peels on Some Blood Serum Biochemical Parameters in Alloxan Induced Diabetes Male Rats.” Tikrit Journal for Agricultural Sciences 21, no. 1: 138–151. 10.25130/tjas.21.1.14.

[fsn371411-bib-0025] Ketuly, K. A. , M. A. Abdulla , H. A. Hadi , A. A. Mariod , and S. I. Abdel‐Wahab . 2011. “Anti‐Ulcer Activity of the 9alphabromo Analogue of Beclomethasone Dipropionate Against Ethanol‐Induced Gastric Mucosal Injury in Rats.” Journal of Medicinal Plant Research 5, no. 4: 514–520.

[fsn371411-bib-0026] Krzyżek, P. , P. Migdał , E. Paluch , M. Karwańska , A. Wieliczko , and G. Gościniak . 2021. “Myricetin as an Antivirulence Compound Interfering With a Morphological Transformation Into Coccoid Forms and Potentiating Activity of Antibiotics Against *Helicobacter pylori* .” International Journal of Molecular Sciences 22, no. 5: 2695. 10.3390/ijms22052695. 33800082 PMC7962197

[fsn371411-bib-0027] Liu, C. , H. Guo , N. A. DaSilva , et al. 2019. “Pomegranate ( *Punica granatum* ) Phenolics Ameliorate Hydrogen Peroxide‐Induced Oxidative Stress and Cytotoxicity in Human Keratinocytes.” Journal of Functional Foods 54: 559–567. 10.1016/j.jff.2019.02.015. 34079588 PMC8168454

[fsn371411-bib-0028] Lu, J. , and Q. Yuan . 2008. “A New Method for Ellagic Acid Production From Pomegranate Husk.” Journal of Food Process Engineering 31: 443–454.

[fsn371411-bib-0030] Man, G. , L. Xu , Y. Wang , X. Liao , and Z. Xu . 2022. “Profiling Phenolic Composition in Pomegranate Peel From Nine Selected Cultivars Using UHPLC‐QTOF‐MS and UPLC‐QQQ‐MS.” Frontiers in Nutrition 8: 807447. 10.3389/fnut.2021.807447. 35141267 PMC8819070

[fsn371411-bib-0031] Mayyas, A. , M. Abu‐Sini , R. Amr , et al. 2021. “Novel In Vitro and In Vivo Anti‐ *Helicobacter pylori* Effects of Pomegranate Peel Ethanol Extract.” Veterinary World 14, no. 1: 120–128. 10.14202/vetworld.2021.120-128. 33642795 PMC7896906

[fsn371411-bib-0032] Mo, Y. , J. Ma , W. Gao , et al. 2022. “Pomegranate Peel as a Source of Bioactive Compounds: A Mini Review on Their Physiological Functions.” Frontiers in Nutrition 9: 887113. 10.3389/fnut.2022.887113. 35757262 PMC9218663

[fsn371411-bib-0033] Mohamed, M. S. , and H. Mabrok . 2022. “Protective Effect of Pomegranate Peel Powder Against Gastric Ulcer in Rats.” Biointerface Research in Applied Chemistry 12, no. 4: 4888–4899. 10.33263/BRIAC124.48884899.

[fsn371411-bib-0034] Mohan Prasad, V. G. , L. V. McFarland , H. P. Thacker , R. Puri , and P. S. Lawate . 2024. “Efficacy and Safety of Omeprazole for the Treatment of Acid Peptic Disorders: A Systematic Review and Meta‐Analysis.” International Journal of Clinical Practice 2024: 9990554. 10.1155/2024/9990554.

[fsn371411-bib-0035] Monika, P. , M. N. Chandraprabha , R. Hari Krishna , et al. 2022. “Recent Advances in Pomegranate Peel Extract Mediated Nanoparticles for Clinical and Biomedical Applications.” Biotechnology and Genetic Engineering Reviews 40, no. 4: 3379–3407. 10.1080/02648725.2022.2122299. 36117472

[fsn371411-bib-0036] Muhialdin, A. J. , Z. Z. Alamri , A. M Hussein , et al. 2023. “Gastro‐Protective and Therapeutic Effect of *Punica granatum* Against Stomach Ulcer Caused by *Helicobacter Pylori* .” Cellular and Molecular Biology (Noisy‐le‐Grand, France) 69, no. 1: 48–53. 10.14715/cmb/2022.69.1.9. 37213156

[fsn371411-bib-0037] Nabil, M. , M. A. El Raey , W. Abdo , et al. 2021. “Gastro‐Protective Effects of Albizia Anthelmintica Leaf Extract on Indomethacin‐Induced Gastric Ulcer in Wistar Rats: In Silico and In Vivo Studies.” Antioxidants (Basel) 10, no. 2: 176. 10.3390/antiox10020176. 33530540 PMC7911069

[fsn371411-bib-0038] Rudra, D. S. , U. Pal , N. Chowdhury , N. C. Maiti , A. Bagchi , and S. Swarnakar . 2022. “Omeprazole Prevents Stress Induced Gastric Ulcer by Direct Inhibition of MMP‐2/TIMP‐3 Interactions.” Free Radical Biology & Medicine 181: 221–234. 10.1016/j.freeradbiomed.2022.02.007. 35150824

[fsn371411-bib-0039] Sahebkar, A. , C. Ferri , P. Giorgini , S. Bo , P. Nachtigal , and D. Grassi . 2017. “Effects of Pomegranate Juice on Blood Pressure: A Systematic Review and Meta‐Analysis of Randomized Controlled Trials.” Pharmacological Research 115: 149–161. 10.1016/j.phrs.2016.11.018. 27888156

[fsn371411-bib-0040] Sayanti, B. , R. C. Susri , C. Subrata , and K. B. Sandip . 2007. “Healing Properties of Some Indian Medicinal Plants Against Indomethacin‐Induced Gastric Ulceration of Rats.” Journal of Clinical Biochemistry and Nutrition 41, no. 2: 106–114. 10.3164/jcbn.2007015. 18193104 PMC2170955

[fsn371411-bib-0041] Sayed, S. , S. S. Alotaibi , A. M. El‐Shehawi , et al. 2022. “The Anti‐Inflammatory, Anti‐Apoptotic, and Antioxidant Effects of a Pomegranate‐Peel Extract Against Acrylamide‐Induced Hepatotoxicity in Rats.” Life 12, no. 2: 224. 10.3390/life12020224. 35207511 PMC8878900

[fsn371411-bib-0042] Serafim, C. , M. E. Araruna , E. A. Júnior , M. Diniz , C. Hiruma‐Lima , and L. Batista . 2020. “A Review of the Role of Flavonoids in Peptic Ulcer (2010‐2020).” Molecules 25: 5431. 10.3390/molecules25225431. 33233494 PMC7699562

[fsn371411-bib-0043] Sheir, M. , and R. Ahmed . 2022. “Role of Some Types of Bee's Honey in Gastric Ulcer Healing by Regulating HSP47 and VEGF Expression.” Egyptian Journal of Chemistry 65, no. 7: 385–408. 10.21608/ejchem.2021.104417.4831.

[fsn371411-bib-0044] Silla, A. , A. Punzo , F. Bonvicini , et al. 2025. “Anti‐Inflammatory, Antioxidant and Antibacterial Properties of Tomato Skin and Pomegranate Peel Extracts: A Sustainable Approach for Oral Health Care.” Antioxidants 14, no. 1: 54. 10.3390/antiox14010054. 39857388 PMC11762152

[fsn371411-bib-0045] Singh, B. , J. P. Singh , A. Kaur , and N. Singh . 2018. “Phenolic Compounds as Beneficial Phytochemicals in Pomegranate ( *Punica granatum* L.) Peel: A Review.” Food Chemistry 261: 75–86. 10.1016/j.foodchem.2018.04.039. 29739608

[fsn371411-bib-0046] Sisto, F. , M. M. Scaltrito , C. Masia , et al. 2016. “In Vitro Activity of Artemisone and Artemisinin Derivatives Against Extracellular and Intracellular *Helicobacter pylori* .” International Journal of Antimicrobial Agents 48, no. 1: 101–105. 10.1016/j.ijantimicag.2016.03.018. 27216383

[fsn371411-bib-0047] Takagi, K. , S. Okabe , and R. Saziki . 1969. “A New Method for the Production of Chronic Gastric Ulcer in Rats and the Effect of Several Drugs on Its Healing.” Japanese Journal of Pharmacology 19, no. 3: 418–426. 10.1254/jjp.19.418. 5307474

[fsn371411-bib-0048] Tang, L. , L. Liu , L. Sun , Y. Qin , J. Li , and X. Li . 2010. “Extraction and Composition Analysis of Polyphenols in Pomegranate Skin.” Journal of Food Research and Development 2010, no. 5: 121–126.

[fsn371411-bib-0049] Turrini, E. , L. Ferruzzi , and C. Fimognari . 2015. “Potential Effects of Pomegranate Polyphenols in Cancer Prevention and Therapy.” Oxidative Medicine and Cellular Longevity 2015: 938475. 10.1155/2015/938475. 26180600 PMC4477247

[fsn371411-bib-0050] Vučić, V. , M. Grabež , A. Trchounian , and A. Arsić . 2019. “Composition and Potential Health Benefits of Pomegranate: A Review.” Current Pharmaceutical Design 25, no. 16: 1817–1827. 10.2174/1381612825666190708183941. 31298147

[fsn371411-bib-0051] Wang, G. Z. , G. P. Huang , G. L. Yin , et al. 2007. “Aspirin Can Elicit the Recurrence of Gastric Ulcer Induced With Acetic Acid in Rats.” Cellular Physiology and Biochemistry: International Journal of Experimental Cellular Physiology, Biochemistry, and Pharmacology 20, no. 1–4: 205–212. 10.1159/000104167. 17595529

[fsn371411-bib-0052] Zamanian, M. Y. , Z. R. Gardanova , A. Hjazi , et al. 2025. “Pomegranate as a Natural Remedy for Gastric Ulcers Prevention: A Review of Its Gastroprotective Mechanisms and Pharmacological Benefits.” Naunyn‐Schmiedeberg's Archives of Pharmacology 398, no. 6: 6675–6690. 10.1007/s00210-025-03822-8. 39888366

